# Premature oral pre-shaping for feeding in elderly population with risk of aspiration pneumonia

**DOI:** 10.1371/journal.pone.0246804

**Published:** 2021-02-08

**Authors:** Yoshiki Tamaru, Akiyoshi Matsugi, Shinzo Masaki, Yoshihito Tsubouchi, Akiyoshi Yanagawa

**Affiliations:** 1 Faculty of Rehabilitation, Shijonawate Gakuen University, Daito, Osaka, Japan; 2 Dementia Care at Home, Nobana Healthpromote, Kishiwada, Osaka, Japan; 3 Faculty of Health Sciences, Naragakuen University, Nara, Nara, Japan; 4 Department of Rehabilitation, Tesseikai Neurosurgical Hospital, Shijonawate, Osaka, Japan; Clinca Geriatrica, ITALY

## Abstract

The aim of this study was to determine the abnormal hand and mouth behavior before actual swallowing for eating in elderly people with high risk of aspiration pneumonia. Ten elderly people with a diagnosis of aspiration pneumonia (EAP), 15 healthy elderly (HE) people, and 21 young adults (YA) were enrolled. The feeding time and the timing of the maximum distance between the upper and lower lips were extracted using a motion analyzer during self-feeding and assisted-feeding. The results showed that feeding time in EAP was significantly longer than that for the other groups in self- and assisted-feeding. In self-feeding, the timing of mouth-preparation in the EAP group was significantly earlier than that in the other groups; conversely, in assisted-feeding, the timing in EAP was significantly delayed. Our results indicate that abnormal preparation of mouth-shape and movement time of hand before actual swallowing in both self- and assisted-feeding may exist in elderly people with previous experience of aspiration pneumonia.

## Introduction

Aspiration pneumonia (AP) is an important problem that needs to be solved, because in Japan, which has a significant aging population, more than 70% hospitalizations for pneumonia are due to aspiration pneumonia [1]. These AP cases have been reported to be at an increased risk of developing cerebrovascular disease [2–4], Parkinson’s disease [5, 6], dementia [7, 8] and age in the elderly [9–13]. The causes of AP are reported as the proliferation of oral bacteria due to subclinical aspiration, or the accidental ingestion of food or liquids [1, 14]. Therefore, accidental entry of bacteria-laden saliva or food through the airway and into the lungs is thought to be a major factor. Subsequently, decrease of swallowing ability can lead to AP [1, 14].

For assessment of the swallowing ability, the swallowing process can be divided into five stages: (1) the pre-oral (anticipatory) phase, (2) the preparatory phase, (3) the oral phase, (4) the pharyngeal phase, and (5) the esophageal phase [15]. The mouth and tongue movement are involved in phases 1–3 for preparation of the 4th phase (pharyngeal phase) where swallowing failure is likely to occur. Videofluorography [16, 17], video endoscopy [18], and the 100-mL water swallow test [19, 20] examination are the gold standards for assessment of the pharyngeal phase. However, for the pre-oral and preparation phase, no standard assessment methods are currently available.

Recently, many studies have reported that the pre-oral stage is involved in swallowing function [21–26]. Visual food cognition modulates the oscillatory brain activity related to swallowing during the anticipatory stage of swallowing [22]. A drink-related visual stimulus facilitates the initiation of voluntary swallowing [23, 24]. Olfactory sensitivity [25] and saliva secretion in the pre-oral phase are involved in the swallowing function [26]. These studies indicate that cognitive and oral preparation are important for successful swallowing. Furthermore, the pre-shaping of the hand before gripping depends on visual information, indicating that it reflects the course of subsequent movement [27, 28]. Based on these findings, we hypothesized that abnormal behavior appears in pre-shaping of the lip for eating in elderly people with experience of AP, who may have dysfunctions associated with aspiration. Therefore, in this study, we compared the timing of preparation of the lip for feeding in elderly people with and without AP experience to investigate the relationship between abnormal mouth behavior and aspiration. To further examine the impact of aging on oral preparation, we also compared young and older adults with no AP experience.

Grasping an object by hand provides information about its size, weight, shape, and hardness. This offers more information for oral preparation than the acquisition of information by sight and smell alone. Furthermore, if you can bring food to your mouth using your own hands, you are more likely to be able to successfully time the opening of the mouth. In other words, the abnormality of the preparation process is more pronounced when food is delivered by others, and information is acquired only by sight and smell. Therefore, we also investigated whether the abnormality of the behavior is amplified through self- and assisted-feeding conditions.

In summary, to investigate the relationship of abnormal lip movement in the pre-oral phase for feeding and aspiration, we compared the timing of maximum lip movement between young adults, healthy elderly, and elderly with experience of AP using self- and assisted-feeding tasks.

## Materials and methods

### Participants

Twenty-one young adults (13 males and 8 females; mean age 21.8±0.4 years; YA), 15 healthy elderly individuals (6 males and 9 females; mean age 74.9±6.2 years; Mini-Mental State Examination [MMSE] 26.2±6.2; HE), and 10 HE at high risk for AP (4 males and 6 females; mean age 78.0±4.5 years; MMSE 25.3±1.6; EAP) participated this study. Table 1 shows the attributes. The definition of EAP in this study was more than two diagnoses of EAP in 2019–2020. Criteria for rejection of the participants were as follows: presence of dementia, cerebrovascular disease, and other disorders that would hinder the study. All participants were informed of the aim of the study and provided signed informed consent before participation following guidelines approved by the Shijonawate-Gakuen University of Faculty of Rehabilitation research ethics review committee (Approval No. 19–2), and this study was conducted in accordance with the Declaration of Helsinki.

**Table 1:** Attributes.

	YA	HE	EAP	p-value
n	21	15	10	-
Sex	M: 13 F: 8	M: 6 F: 9	M:4 F: 6	-
Age (years)	21.8± 0.4	74.9± 6.2	78.0±4.5	YA vs. HE p = 0.001
				YA vs. EAP p = 0.001
				HE vs. EAP p = 0.09
MMSE	-	26.2±1.5	25.3±1.6	p = 0.180
Dominant hand (by EHI)	R: 21 L: 0	R: 14 L: 1	R: 9 L: 1	-

YA = young adult, EAP = aspiration pneumonia.

R = Right hand, L = Left hand, EHI = Edinburgh handedness inventory.

Kruskal-Wallis test was used for age. Mann-Whitney test was used for MMSE.

### Experimental design

**Fig 1:**
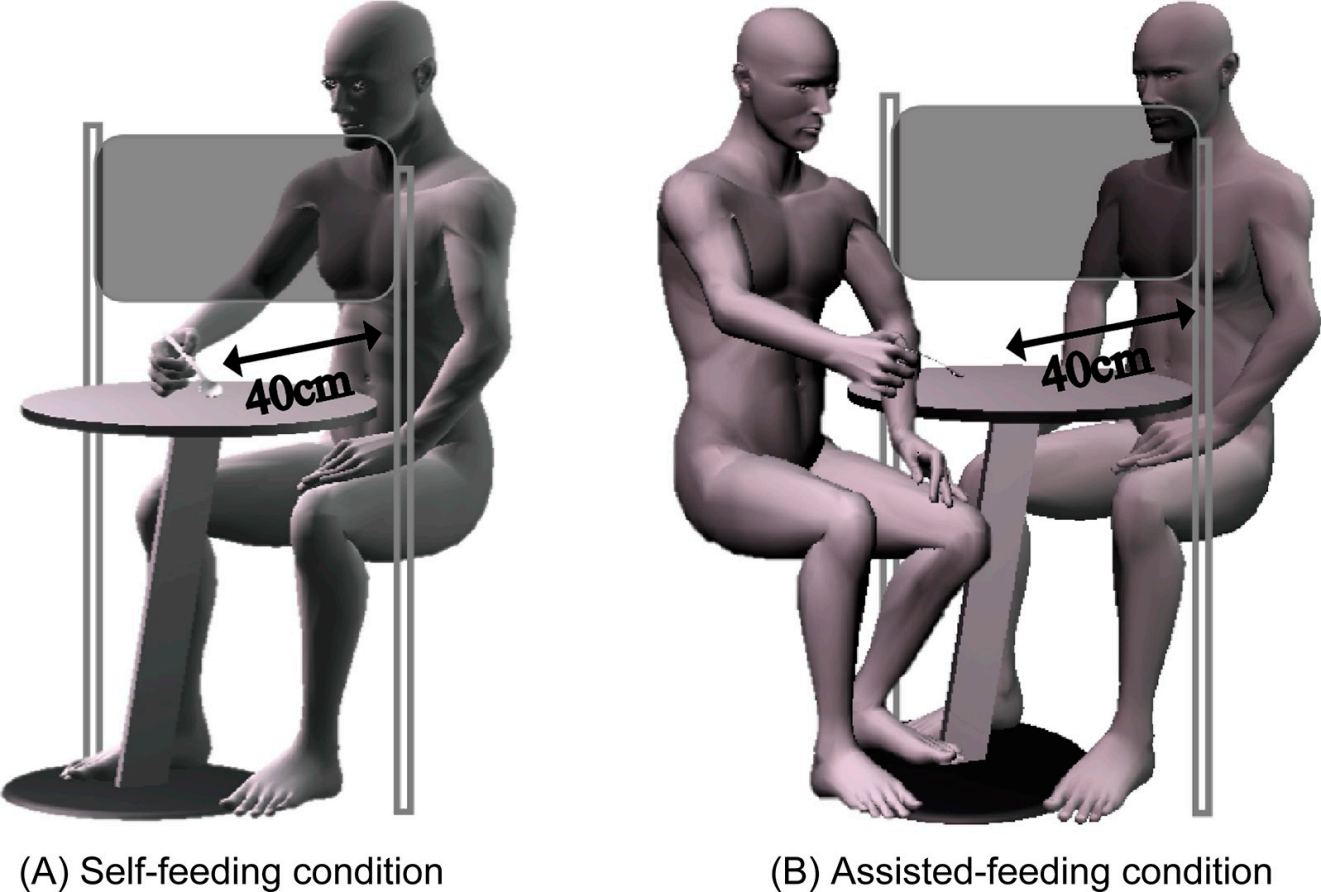
Environment settings and each condition. (A) Self-feeding condition was usual. (B) Assistance by others for feeding. In both conditions, the board was placed in front of the subject so that they could not perceive the food by visual information before eating.

The Edinburgh Handedness Inventory [29] was used to determine the dominant hand. The measurement environment was a sitting position. A spoon with a bite-sized rice porridge that was usually eaten was gripped with the dominant hand. As for the starting posture, the position of the spoon was set 40 cm forward of the abdomen as shown in Fig 1. The tasks were self-feeding and assisted-feeding. Self-feeding conditions were the subject’s usual eating method. The assisted-feeding condition was a method of eating assisted by others. The board was placed in front of the subject so as to not perceive the food visually before eating. The subject ate immediately after removing the board. The subject was not informed of the kind of food he/she was about to eat until the start of feeding. A one-week washout period was provided for the implementation of the self-feeding condition and the assisted-feeding condition.

### Feeding time & oral pre-shaping measurements

**Fig 2:**
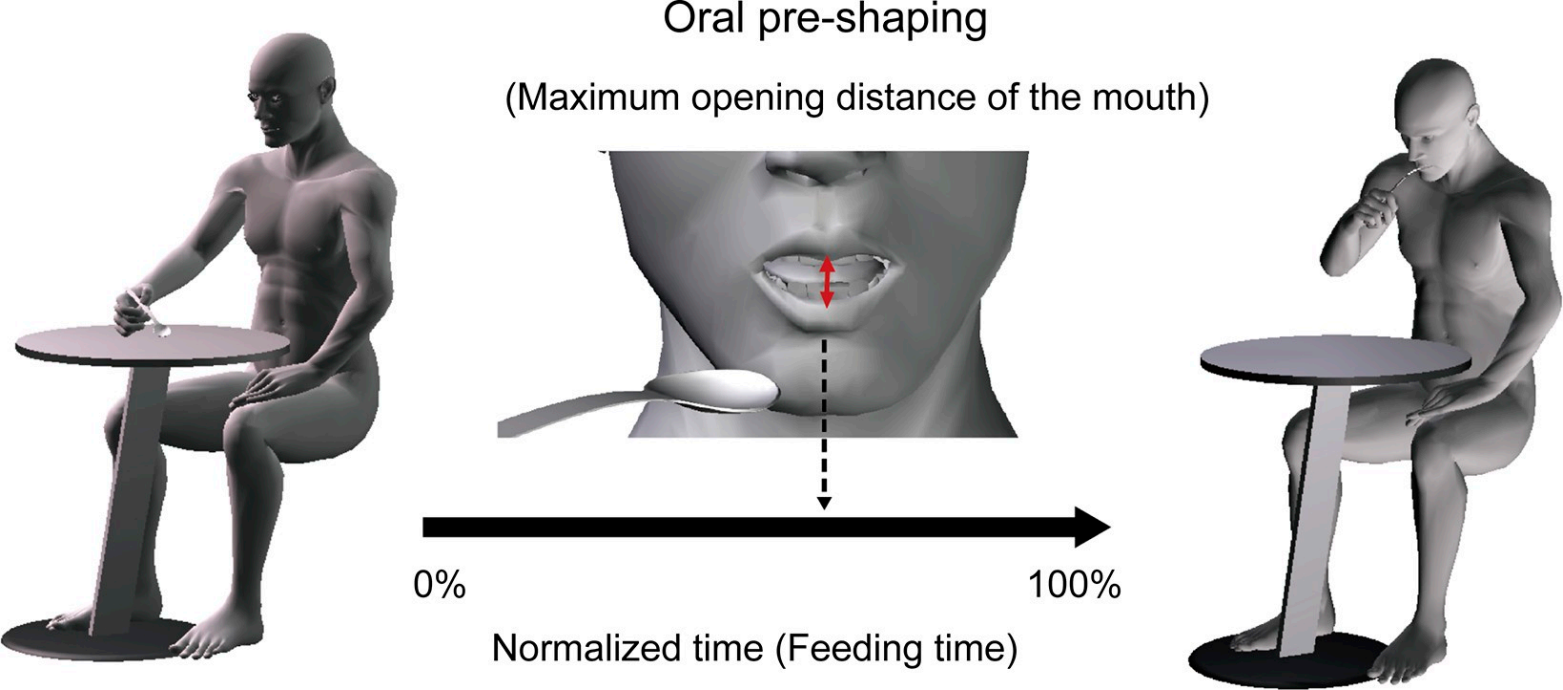
Feeding time & oral pre-shaping measurements. Oral pre-shaping was defined as the time of maximum opening distance of the mouth by normalized feeding time.

In the analysis of oral pre-shaping, the period from the movement of the spoon to the ingestion into the oral cavity was recorded with a video camera (HC-WXF990M 60 Hz Panasonic Inc., Japan). The recorded data was used for calculation, by a motion analysis software [Dartfish analyzer (Dartfish Inc., Japan)], of feeding time and opening distance of the mouth besides the maximum opening distance point of the mouth for normalized feeding time (Fig 2). In this study, oral pre-shaping was defined as the maximum opening distance of the mouth between the upper and lower lips during feeding.

### Statistical analysis

Statistical analysis was done using Statistical Package for Social Sciences Version 17 (SPSS Inc., USA). For non-parametric data, the Shapiro-Wilk test was used. Kruskal-Wallis test and post hoc test (Mann-Whitney U test) were used to compare the differences in feeding time and oral pre-shaping for each group in both conditions. The Wilcoxon signed-rank test was used to compare the differences in groups. The significance level was set at *p*<0.05.

## Results

### Feeding time

**Fig 3:**
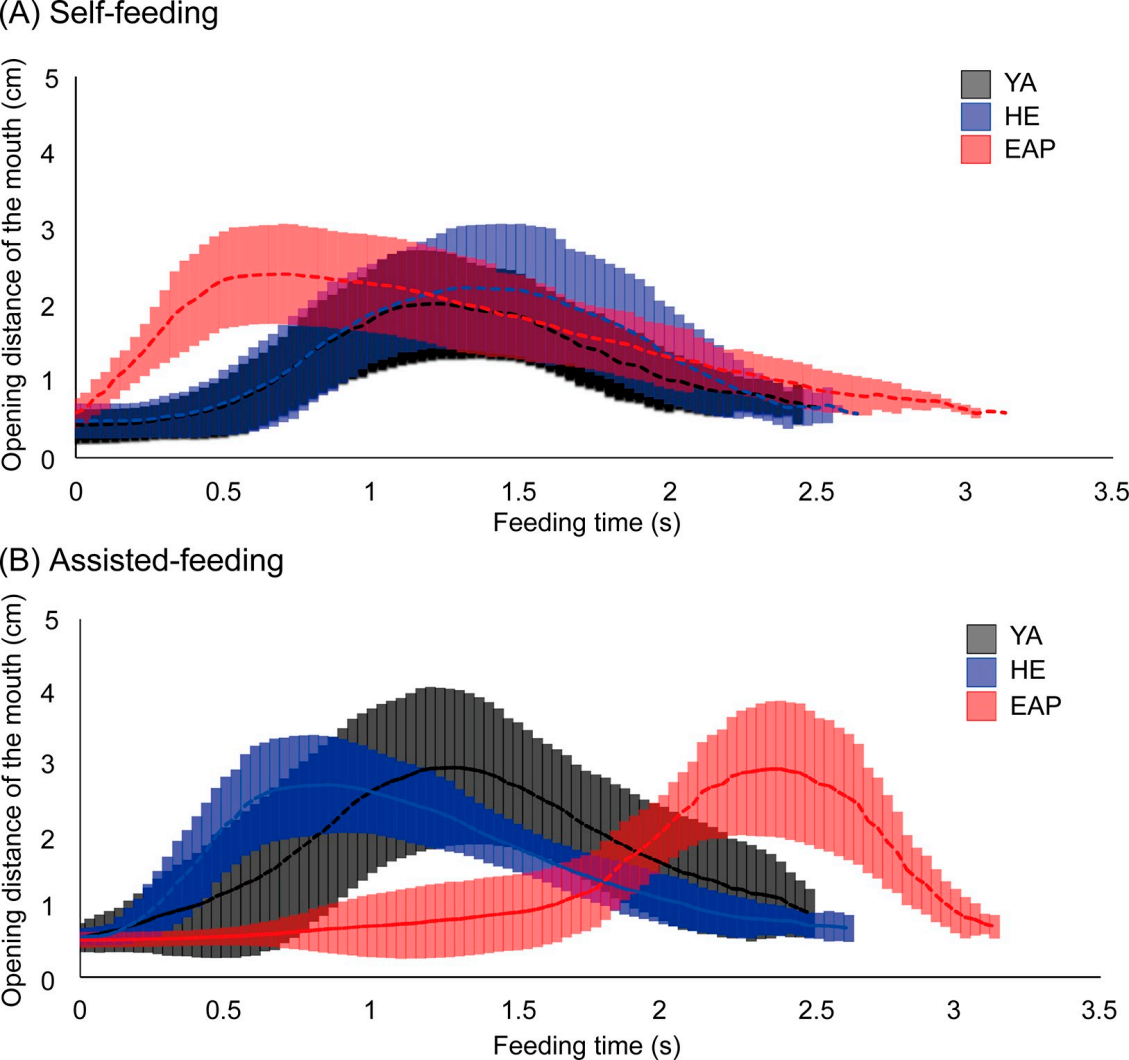
Opening distance of the mouth and feeding time. (A) and (B) both show the oral distance to feeding time. The middle line is the average of each subject, and the top and bottom indicate the standard deviation. The vertical axis indicates the opening distance between the upper lip and lower lip (in cm). The horizontal axis shows the time from the start of feeding until the food is taken into the oral cavity (in s). The black line indicates a healthy young adult (YA), the blue line indicates an HE adult, the red line indicates an elderly individual with aspiration pneumonia (EAP).

**Fig 4:**
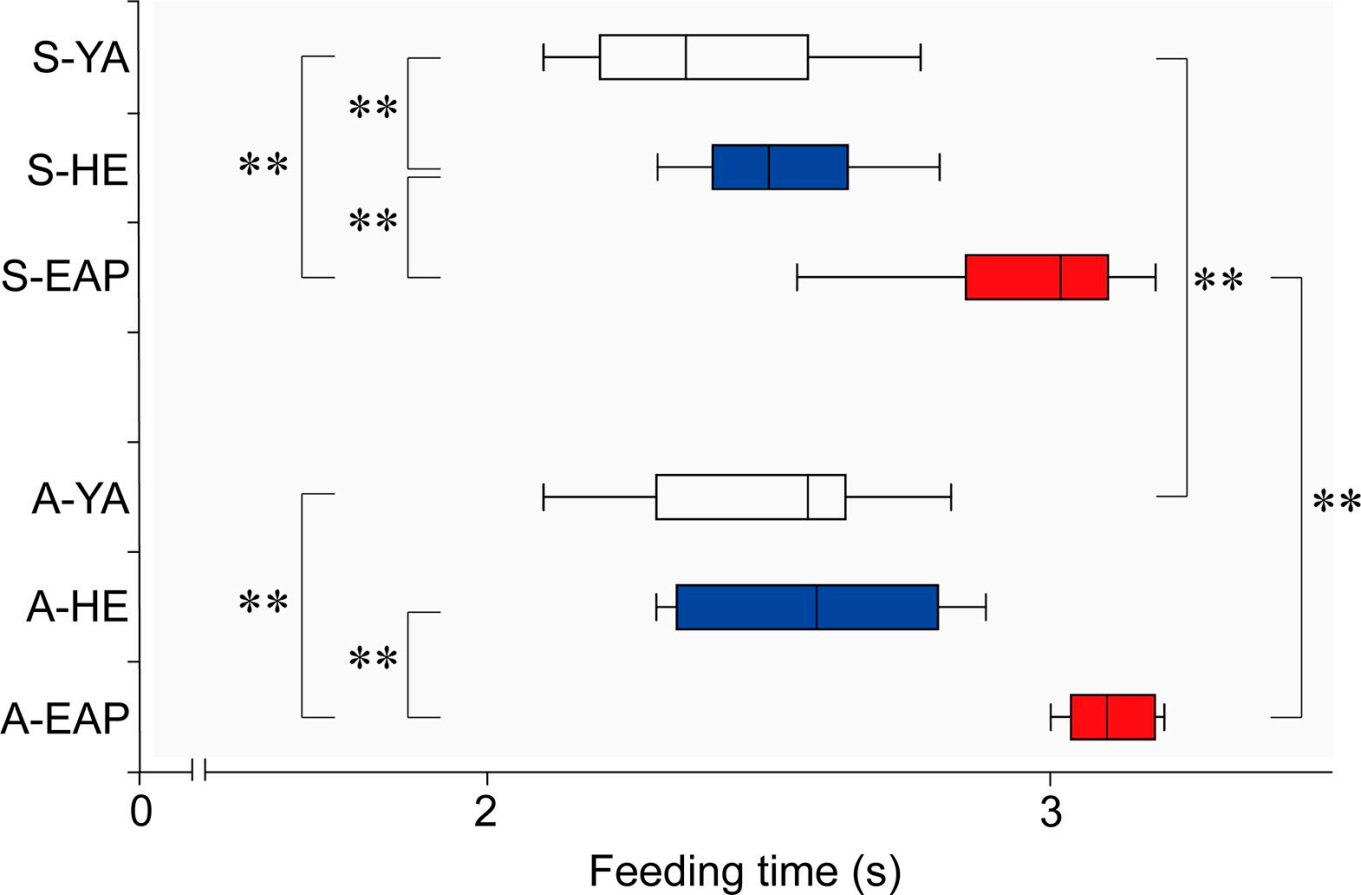
Feeding time. The vertical axis indicates the condition and group pairs. The horizontal axis indicates the feeding time (in s). Box whisker plot shows median (minimum -25% -75% -maximum). Asterisks signify differences (*p<0.05, **p<0.01). S- = self-feeding, A- = assisted-feeding. YA = young adult, EAP = elderly individual with aspiration pneumonia.

The results are shown in Figs 3 and 4, and S1 Table. In self-feeding, with respect to YA [median (25–75%); 2.27 s (2.17–2.40 s)], HE [2.50 s (2.40–2.52 s)], and EAP [2.82 s (2.64–2.98 s)], feeding time in EAP was significantly delayed than in YA and HE. In assisted-feeding, with respect to YA [2.57 s (2.30–2.64 s)], HE [2.57 s (2.37–2.75 s)], and EAP [3.09 s (3.04–3.16 s)], EAP was the slowest among the three-groups. In the intra-group comparisons, HE did not show a significant difference between self-feeding and assisted-feeding. For YA and EAP, assisted-feeding was significantly delayed compared to self-feeding.

**Fig 5:**
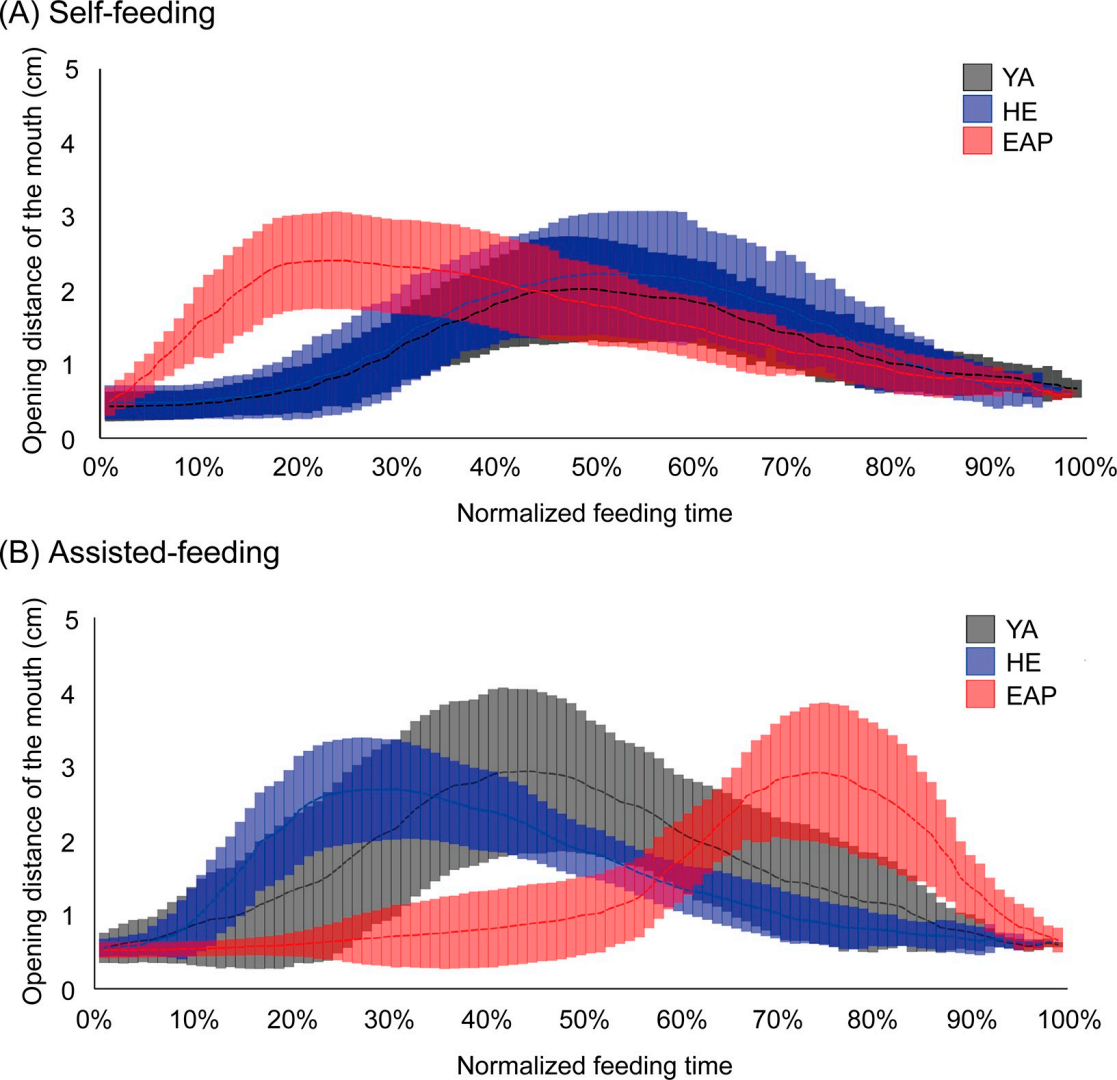
Opening distance of the mouth and normalized feeding time. (A) and (B) show the opening distance of the mouth and the feeding time for normalization of the feeding time. The middle line is the average for each subject, and the top and bottom lines indicate the standard deviation. The vertical axis indicates the opening distance of the mouth between the upper lip and lower lip (in cm). The horizontal axis indicates the normalized feeding time from the start of feeding until the food was taken into the oral cavity. The black line indicates a healthy young adult (YA), the blue line indicates a HE adult, the red line indicates an elderly individual with aspiration pneumonia (EAP).

**Fig 6:**
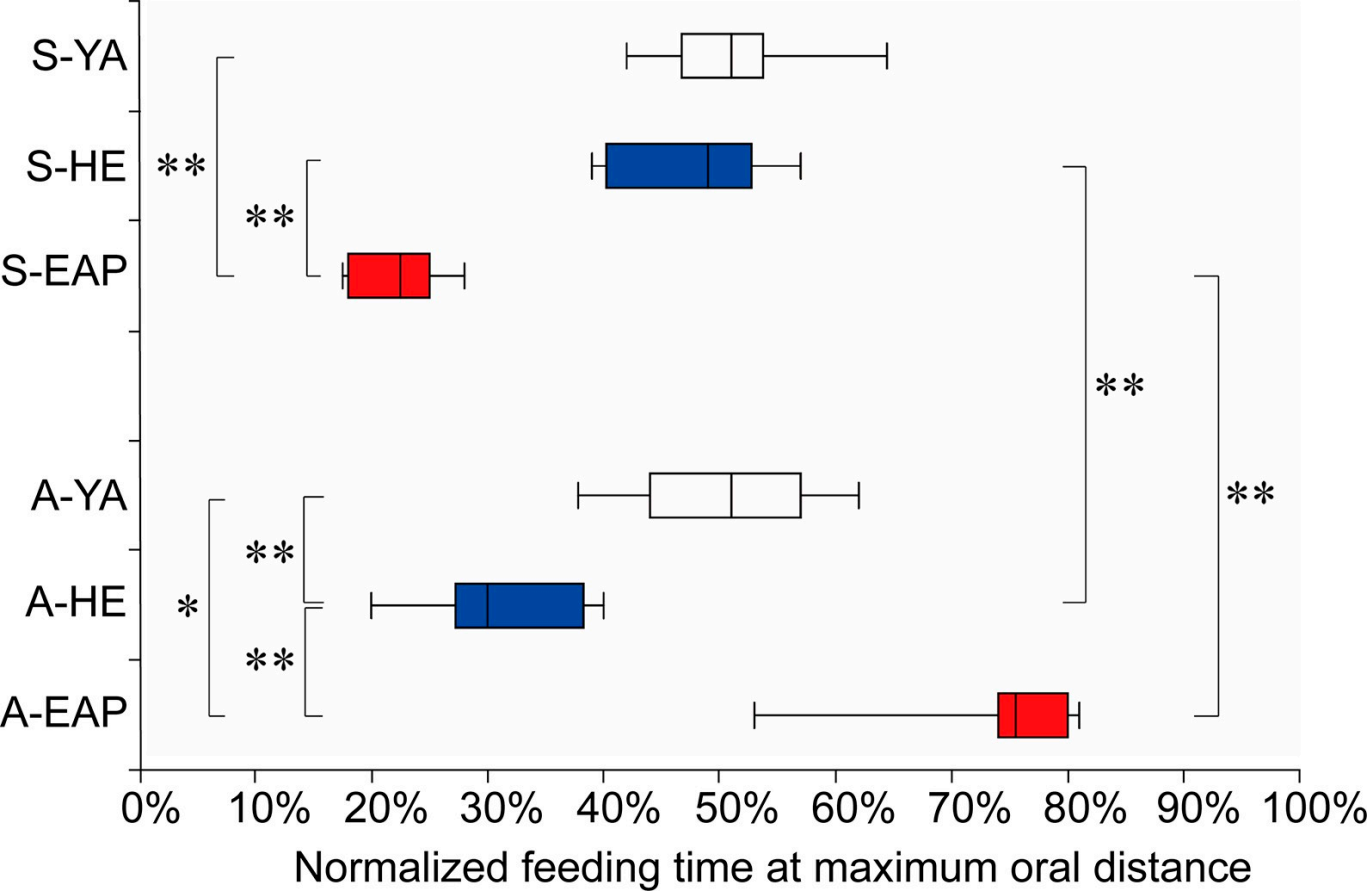
Oral pre-shaping. The vertical axis indicates the condition and group pairs. The horizontal axis indicates the normalized the feeding time. Box whisker plot shows median (minimum -25% -75% -maximum). Asterisks indicate significant differences (*p<0.05, **p<0.01). S- = self-feeding, A- = assisted-feeding. YA = young adult, EAP = elderly individual with aspiration pneumonia.

The results are shown in Figs 5 and 6, and S2 Table. In self-feeding, with respect to YA [50.8% (47.0–53.1%)], HE [50.6% (41.0–53.5%)], and EAP [22.6% (18.2–24.8%)], there was no significant difference between YA and HE. Feeding time in EAP was significantly shorter than in both groups. 2) In assisted-feeding, with respect to YA [50.9% (44.2–56.5%)], HE [29.9% (27.6–37.5%)], and EAP [75.1% (74.0–80.4%)], the time was significantly shorter in HE than in YA. Besides, EAP was significantly delayed compared to both groups. In the in-group comparisons, YA did not differ significantly in oral pre-shaping in self-feeding and assisted-feeding, but in HE, assisted-feeding time was significantly shorter than self-feeding. In EAP, on the contrary, assisted-feeding was significantly delayed.

## Discussion

The purpose of this study was to clarify the characteristics of the pre-oral (anticipatory) phase of the swallowing process in healthy HE people and HE people at high risk for AP. A cross-sectional study was performed under two conditions: self-feeding and assisted-feeding. In the pre-oral (anticipatory) phase, the feeding time and time when the mouth had maximal opening distance (oral pre-shaping) were determined by normalizing the feeding time. The hypothesis of this study was that EAP had delayed oral pre-shaping due to inadequate preparation of both self-feeding and assisted-feeding in the preceding period. According to the results, feeding time values showed that the EAP group was significantly slow in both self-feeding and assisted-feeding in comparison to the YA and HE groups. In-group comparison showed that the HE group did not have a significant difference between self-feeding and assisted-feeding, but in EAP, assisted-feeding was significantly delayed compared to self-feeding. In oral pre-shaping, EAP self-feeding was significantly faster than in both the groups. However, assisted-feeding in EAP was significantly delayed than both the groups. In the intra-group comparison, YA had no significant difference in oral pre-shaping between self-feeding and assisted-feeding, but HE had significantly faster assisted-feeding than self-feeding. In contrast, the EAP showed a significant delay in assisted-feeding. These findings indicate that the EAP may have already expressed the abnormality from the time of mouth opening before food enters the mouth.

### The difference in feeding time between self-feeding and assisted-feeding

In self-feeding, feeding time in HE was longer than in YA. Age differences between the two groups may indicate that aging affected the speed at which food was transported. Aging reduces the performance and time of movement required in daily life [30]. It is thought that this delay in performance time is affected by muscle weakness [31–33] and decreased cutaneous sensation [34–37]. Participants in this study were independent in their daily life, but it might be affected by subclinical age-appropriate muscle weakness and decreased cutaneous sensation. On the other hand, the EAP group had a more significant delay than the HE group. There was no significant difference in age and MMSE between the EAP group and HE group. These results suggest that the difference in feeding time between the two groups may not be due to aging or cognitive function. The significant difference in oral pre-shaping, differences in the process of an oral preparation of eating may be one of the causes of the difference in feeding time.

In assisted-feeding, only the feeding time in the EAP was significantly prolonged. Under the assisted-feeding condition, the caregiver confirmed that the subject opens the mouth before carrying food to the mouth, so that the feeding time delay in the EAP may be due to the delay in the opening. These results suggest that feeding time may be prolonged in HE people at high risk of AP because both self-feeding and assisted-feeding are prolonged in the oral preparation process before swallowing.

### Oral pre-shaping

#### HE mouth opening is faster in assisted- than in self-feeding

In the assisted-feeding condition, the HE group had significantly earlier oral pre-shaping than the YA group, but there was no significant difference between the two groups under the self-feeding condition. These results suggest that the HE are ready to take food quickly into the mouth when others bring food to their mouth. Such premature movement preparation has been observed in the HE [27, 28, 38]. Premature movement preparation is observed in the task of predicting movement and matching the body. These are thought to be movements that compensate for the feedforward error associated with aging [27, 28, 38]. As a result of assisted-feeding in this study, earlier mouth opening in older adults may be due to a similar compensation mechanism. On the other hand, it was shown that the time of the EAP group was significantly longer under the feeding assistance condition. However, oral pre-shaping was faster in self-feeding, and this is not considered to be due to a decrease in oral movement speed. It was thought that the timing of the healthy HE was delayed due to the inability to prepare for premature compensation. EAP patients have a delayed timing of the swallowing reflex, but there may be a delay in the cognitive process of oral preparation from visual perception in the preceding period.

#### EAP show faster self-feeding than HE but slower assisted-feeding than other groups

Self-feeding in the EAP group was significantly faster in oral pre-shaping than in the HE group but was significantly delayed in assisted-feeding than the other groups. In self-feeding, the perception of the shape, size, and viscosity of food is easy to understand through visual information and self-operation of spoon. However, since only assisted-feeding provides visual information, perceptual information such as differences in food quality may have caused a difference in preparation of the oral cavity in the preceding period of swallowing.

This experiment did not reveal the cognitive processes involved in the feeding time or oral pre-shaping abnormalities. Dysphagia has been reported to be affected by dementia [39–41]. However, in this study, there was no significant difference in MMSE between the HE group and EAP group. The difference in oral pre-shaping in assisted-feeding was not related to orientation, working memory, delayed recollection, understanding, and spatial perception as assessed by the MMSE.

Therefore, in future, we aim to create a detailed classification of aging-associated cognitive decline (reduced memory/learning, reduced attention/concentration, reduced thinking, reduced linguistic ability, reduced visual space cognition, etc.) [42]. It is necessary to study whether these are involved in feeding time or oral pre-shaping abnormalities.

#### Relationship between AP and oral pre-shaping

The limitation of this study was the inability to establish a clear relationship between abnormal preparation of mouth for eating and the definitive cause of AP, because we had no data regarding the cause of AP, we could not analyze the relationship. AP is caused by the proliferation of oral bacteria due to subclinical aspiration, or the accidental ingestion of food or consumed liquids [1, 14]. Based on the cause, the aspiration is classified into two groups; (1) AP and (2) aspiration pneumonitis. AP is bacterial pneumonia caused by silent aspiration. Aspiration pneumonitis is exogenous and is considered to be due to aspiration. However, the decline of swallowing ability is a common cause in all the cases and it is a known that a complete distinction of the causes of all cases is difficult [1, 14]. Regardless of the cause of AP, our study revealed that elderly people who experienced AP had abnormalities in their preparatory behavior for eating. Based on these ideas, we should initiate a further study of the abnormality in preparation of the mouth for eating in each of the causes of AP.

## Conclusion

The results of this study suggest that the pre-oral (anticipatory) phase of the swallowing process is abnormal in HE at high risk for AP, and that the abnormality may appear as the timing of mouth opening. This discovery may contribute to the development of a screening evaluation method that non-invasively predicts dysphagia using feeding time and oral pre-shaping in self-feeding and assisted-feeding.

## Supporting information

S1 Table Feeding time comparisons between and within each group. (DOCX)

S2 Table Oral pre-shaping comparisons between and within each group. (DOCX)
