# Supplementary material for: Premature oral pre-shaping for feeding in elderly population with risk of aspiration pneumonia
Source: PLoS One. 2021 Feb 8;16(2):e0246804. doi: 10.1371/journal.pone.0246804 (PMC7870084; doi:10.1371/journal.pone.0246804)
Supplement: S1 Table — (DOCX) [file pone.0246804.s001.docx]

**S1 Table.** Feeding time comparisons between each group and intra-group

|  | A-YA | S-HE | A-HE | S-EAP | A-EAP |
| --- | --- | --- | --- | --- | --- |
| S-YA | *d*=-0.70  [-1.18, 0.22]  *p*=0.004 | *d*=1.47  [0.7, 2.21]  *p*=0.001 | - | *d*=2.44  [1.45, 3.40]  *p*= 0.001 | - |
| A-YA | - | - | *d*=0.40  [-0.28, 1.06]  *p*=0.248 | - | *d*=2.56  [1.55, 3.55]  *p*=0.001 |
| S-HE | - | - | *d*=-0.488  [-1.02, 0.06]  *p*=0.08 | *d*=1.48  [0.56, 2.37]  *p*=0.001 | - |
| A-HE | - | - | - | - | *d*=2.74  [1.60, 3.84]  *p*=0.001 |
| S-EAP | - | - | - | - | *d*=-0.971  [-1.72, -0.19]  *p*=0.013 |

Effect size Cohen’s *d* [95% Lower and Upper] and *p*-value)

YA= young adult, EAP: aspiration pneumonia.

□= Comparisons between each group, □= Comparison between In-group.

S-= self-feeding, A-= assisted-feeding. YA= young adult, EAP= aspiration pneumonia.
