# Supplementary material for: Premature oral pre-shaping for feeding in elderly population with risk of aspiration pneumonia
Source: PLoS One. 2021 Feb 8;16(2):e0246804. doi: 10.1371/journal.pone.0246804 (PMC7870084; doi:10.1371/journal.pone.0246804)
Supplement: S2 Table — (DOCX) [file pone.0246804.s002.docx]

**S2 Table.** Oral pre-shaping comparisons between each groups and in-groups

|  | A-YA | S-HE | A-HE | S-EAP | A-EAP |
| --- | --- | --- | --- | --- | --- |
| S-YA | *d*=016  [-0.28, 0.59]  *p*=0.479 | *d*=-0.46  [-1.12, 0.22]  *p*=0.186 | - | *d*=-4.24  [-5.55, -2.90]  *p*=0.001 | - |
| A-YA | - | - | *d*=1.97  [-2.77, 1.15]  *p*=0.001 | - | *d*=2.07  [1.13, 2.97]  *p*=0.001 |
| S-HE | - | - | *d*=1.66  [0.86, 2.44]  *p*=0.001 | *d*=-3.63  [-4.93, -2.30]  *p*=0.001 | - |
| A-HE | - | - | - | - | *d*=4.37  [2.87, 5.84]  *p*=0.001 |
| S-EAP | - | - | - | - | *d*=-3.74  [-5.53, -1.92]  *p*=0.001 |

Effect size Cohen’s *d* [95% Lower and Upper] and *p*-value)

YA= young adult, EAP= aspiration pneumonia.

□= Comparisons between each group, □= Comparison between In-group.

S-= self-feeding, A-= assisted-feeding. YA= young adult, EAP= aspiration pneumonia.
